# Supplementary material for: A Stable Rechargeable Aqueous Zn–Air Battery Enabled by Heterogeneous MoS2 Cathode Catalysts
Source: Nanomaterials (Basel). 2022 Nov 18;12(22):4069. doi: 10.3390/nano12224069 (PMC9698408; doi:10.3390/nano12224069)
Supplement: Supplementary file 1 [file nanomaterials-12-04069-s001.zip › nanomaterials-2027719-supplementary.pdf]

Supplementary Material

A Stable Rechargeable Aqueous Zn–Air Battery Enabled by Heterogeneous MoS_2 Cathode Catalysts

Min Wang [1], Xiaoxiao Huang [2], Zhiqian Yu [1], Pei Zhang [3], Chunyang Zhai [2,*], Hucheng Song [1,*], Jun Xu [1] and Kunji Chen [1]

[1] National Laboratory of Solid State Microstructures, School of Electronics Science and Engineering, College of Engineering and Applied Sciences, Nanjing University, Nanjing 210093, China
[2] School of Materials Science and Chemical Engineering, Ningbo University, Ningbo 315211, China
[3] College of Electrical and Information Engineering, Zhengzhou University of Light Industry, Zhengzhou 450002, China
[*] Correspondence: to: zhaichunyang@nbu.edu.cn (C.Z.); hcsong@nju.edu.cn (H.S.)

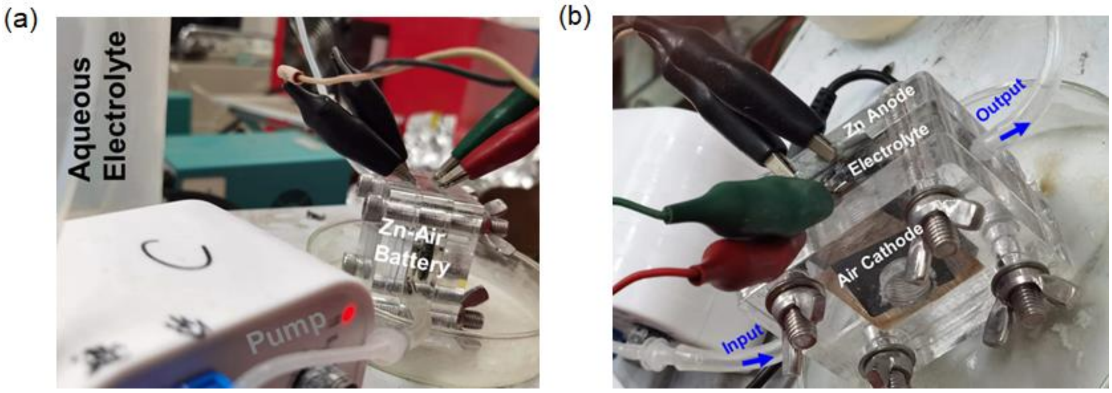

Figure S1. Auqeous Zn-Air battery system. (A) The photo of aquesous Zn-Air battery system that consists of aquesous electrolyte, pump and Zn-Air battery. (B) Photo of aqueous Zn-Air battery that consists of air cathode, aqueous and Zn-anode.

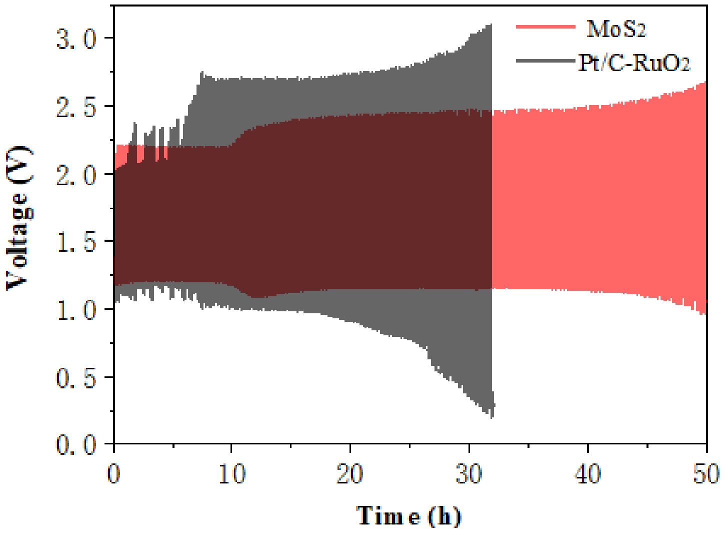

Figure S2. Electrochemical cycle performance of aqueous Zn-Air batteries using the heterogeneous MoS_2 cathode catalyst and commercial Pt/C-RuO_2 cathode catalyst where the Zn-Air battery using the prepared MoS_2 cathode catalysts shows an excellent cycle stability than the Zn-Air battery using expensive Pt/C-RuO_2 cathode catalyst at a large current density of 5 mA cm^{-2}. It indicates that the

heterogeneous MoS_2 catalyst has better oxygen evolution and oxygen reduction catalytic activity ,especailally at operating current desnsity, than commercial Pt/C-RuO_2 catalyst.

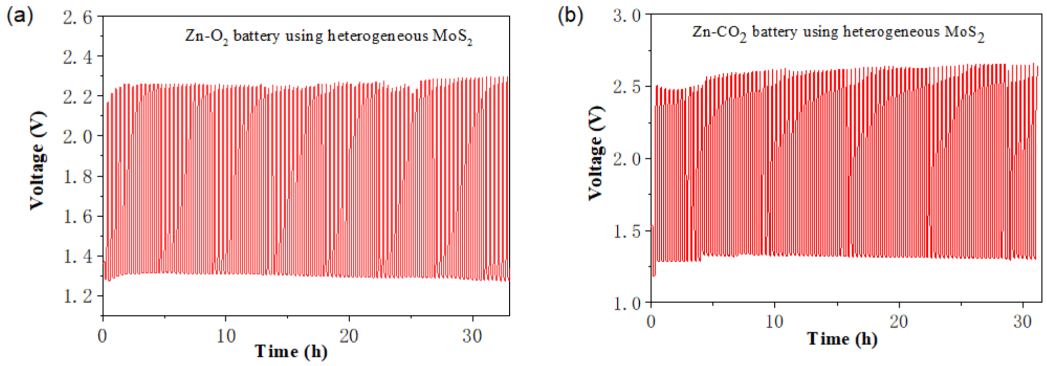

Figure S3. Electrochemical cycle performance of aqueous Zn-O_2 battery and Zn-CO_2 battery. (a) Voltage-time curves of the assembled aqueous Zn-O_2 battery using the heterogeneous MoS_2 catalyst that shows a stable cycle and less than 1.0 V potential gap (with a ~1.3V discharge potential and less than 2.3 V charge potential). (b) Voltage-time curves of the assembled aqueous Zn-O_2 battery using the heterogeneous MoS_2 catalyst that shows a stable cycle and ~1.35 V potential gap (with a ~1.25 V discharge potential and ~ 2.6 V charge potential).

Table S1. Comparison of electrochemical properties of Zn-Air batteries using MoS_2-based catalysts.

Performance / Materials	Capacity [mAh g^{-1}]	Current density [mA cm^{-2}]	Discharge voltage [V]	Charge voltage [V]	Voltage gap [V]	Cycles/Hours	Reference
**MoS_2/PTCDA**	330	0.5	1.30	1.88	0.58	500 cycles/180h	This work
	3300	5	1.22	2.23	1.01	12 h	
**MoS_2 on N-doped carbon**	-	5	1.24	1.99	0.75	48 h	1
**Co_9S_8@MoS_2**	-	10	1.25	2.0	0.75	120 cycles	2
**CoS2@MoS_2@NiS_2**	708	10	1.12	2.07	0.95	40 h	3

The recent reported aqueous Zn-Air batteries are made of bifunctional MoS_2-based catalysts that consist of MoS_2 catalyst and another catalyst with good OER catalytic activity such as N-doped carbon, Co_9S_8 and CoS_2 and NiS_2 (see Ref.1 to Ref.3). The Zn-Air batteries using the heterogeneous MoS_2 catalysts show a long cycle stability (500 cycles, 180 h) and a low discharge voltage (1.88 V) and voltage gap (0.58 V) with a limited capacity of 330 mAh g^{-1}. Even at a limited capacity of 3300 mAh g-1, the Zn-Air battery using the heterogeneous also shows an acceptable oxygen reduction catalytic and oxygen evolution activity (with 1.01 V voltage gap) than the reported bifunctional MoS_2/N-doped carbon,Co_9S_8/MoS_2 and CoS_2/MoS_2/NiS_2 catalysts (with voltage gap from 0.75 V to 0.95 V, see Ref. 1 to Ref 3) with a limited capacity of ~700 mAh g^{-1}.

References

[1] Adv. Funct. Mater.2017, 27, 1702300

[2] ACS Appl. Mater. Interfaces 2018, 10, 2, 1678–1689

[3] Journal of Colloid and Interface Science, 2022(610), 653-662
